# Emotion Self-Regulation in Neurotic Students: A Pilot Mindfulness-Based Intervention to Assess Its Effectiveness through Brain Signals and Behavioral Data

**DOI:** 10.3390/s22072703

**Published:** 2022-04-01

**Authors:** Lila Iznita Izhar, Areej Babiker, Edmi Edison Rizki, Cheng-Kai Lu, Mohammad Abdul Rahman

**Affiliations:** 1 Smart Assistive and Rehabilitative Technology (SMART) Research Group, Electrical and Electronics Engineering Department, Universiti Teknologi PETRONAS, Seri Iskandar 32610, Perak, Malaysia; 2 Computer Engineering Department, Future University, Khartoum 10553, Sudan; areej555@gmail.com; 3 Neuroscience Centre, Universitas Muhammadiyah Prof. Dr. Hamka, Jakarta 12130, Indonesia; rizkiedmiedison@uhamka.ac.id; 4 Department of Electrical Engineering, National Taiwan Normal University, Taipei 106, Taiwan; cklu@ntnu.edu.tw; 5 Faculty of Medicine, Royal College of Medicine Perak, University of Kuala Lumpur, Ipoh 30450, Perak, Malaysia; mohammad@unikl.edu.my

**Keywords:** electroencephalography, mindfulness, emotion regulation, neuroticism

## Abstract

Neuroticism has recently received increased attention in the psychology field due to the finding of high implications of neuroticism on an individual’s life and broader public health. This study aims to investigate the effect of a brief 6-week breathing-based mindfulness intervention (BMI) on undergraduate neurotic students’ emotion regulation. We acquired data of their psychological states, physiological changes, and electroencephalogram (EEG), before and after BMI, in resting states and tasks. Through behavioral analysis, we found the students’ anxiety and stress levels significantly reduced after BMI, with *p*-values of 0.013 and 0.027, respectively. Furthermore, a significant difference between students in emotion regulation strategy, that is, suppression, was also shown. The EEG analysis demonstrated significant differences between students before and after MI in resting states and tasks. Fp1 and O2 channels were identified as the most significant channels in evaluating the effect of BMI. The potential of these channels for classifying (single-channel-based) before and after BMI conditions during eyes-opened and eyes-closed baseline trials were displayed by a good performance in terms of accuracy (~77%), sensitivity (76–80%), specificity (73–77%), and area-under-the-curve (AUC) (0.66–0.8) obtained by k-nearest neighbor (KNN) and support vector machine (SVM) algorithms. Mindfulness can thus improve the self-regulation of the emotional state of neurotic students based on the psychometric and electrophysiological analyses conducted in this study.

## 1. Introduction

Life is getting more and more challenging and often places heavy demands on mental health. University students are no exception. In a recent study, mental health issues were found to be alarming in the student population [1]. A typical student life nowadays, which involves a busy schedule with pressure to perform, is thought to result in decreased efficiency and increased attention problems. Students also experience excessive use of media and a lack of face-to-face communication, especially during the coronavirus disease (COVID-19) pandemic, which can lead to social struggles and impulsive behavior. All these factors can cause the brain state to be constantly in fight or flight, which leads to increased depression and anxiety, also resulting in sleep difficulties. Students with neuroticism are thought to be more negatively affected by all of these. Neurotic individuals are generally thought to have a high temper and less emotional stability [2,3,4,5].

In recent years, there has been an increasing interest in neuroticism within psychology [2,3,4,5,6,7,8,9] due to its robust correlation with a wide variety of physical and mental health problems. Neuroticism is a psychological trait where a person becomes easily irritated with the environment. A neurotic individual will have difficulties in interacting with others, which in turn will lower the individual’s quality of life. Neuroticism can also lead to mental conditions such as anxiety and depression [2,3,4]. It is also associated with an increased risk of physical illnesses such as heart disease, stroke, and digestive problems [5,6]. Besides leading to harmful psychological and physical consequences, neuroticism also prevents the individual from addressing them adequately [6].

It has been suggested that the general population should be screened for neuroticism because it has vast public health implications [5]. During college, neuroticism can negatively affect students’ lives, which then has negative implications on their academic performance. Since the learning age is so critical in a person’s life, it is advisable that the screening for neuroticism starts right then. However, screening without planning how to treat neuroticism would be useless. Hence, treatment strategies need to be in place to help neurotic students improve their quality of life.

There are several methods to improve concentration and increase a sense of calm in neurotic students. Mindfulness is one of these methods and may result in a skillful response to difficult emotions and increased understanding of others, which can help students with neuroticism. Mindfulness has demonstrated a tremendous effect in enhancing human wellbeing, cognitively and emotionally [9,10,11,12,13,14,15,16]. It is also claimed to be effective for a variety of psychological problems, particularly for reducing anxiety, depression, and stress [14]. While some research has studied the effect of meditation on neurotic college students [17], little attention has been given to mindfulness’ effect on similar groups. Mindfulness involves centering attention to present-moment experience non-judgmentally, which can contribute to improvements in attentional control, promote relaxation, and reduce prejudice and cognitive bias. Once relaxation, enhanced awareness, and reduced cognitive biases are gained from mindfulness, this leads to improved emotion regulation processes. Emotion regulation can be aimed at reducing, strengthening, or maintaining the experience that can be associated with either positive or negative emotions, depending on the needs or goals. Emotion regulation is also described as a process targeted at maximizing positive emotions and minimizing negative feelings [15]. It is also a process whereby individuals experiencing an emotional situation try to adjust the type or amount of emotion they are experiencing. In other words, emotion regulation is how humans regulate their feelings and control them to avoid being guided or influenced by emotions, especially negative ones.

Tang et al. showed that mindfulness achieves better stress regulation by a faster decrease in the levels of the stress hormone cortisol following a stressful laboratory task after 5 days of meditation training at 20 min a day, compared to those who received relaxation training [18]. Mindfulness results in changes in the structure and neural patterns present in the brain. Thicker cortical regions related to attention and sensory processing in long-term mindfulness practitioners compared to non-practitioners were reported in [19] using the magnetic resonance imaging (MRI) technique. Many studies have showed positive results in improving emotional mental health among clinical and healthy populations (for a review, see [20]). Brief mindfulness practices have an immediate effect on emotion, mood, anxiety, and improving wellbeing [21,22,23,24]. Current research is heading towards practical and more time-inhibited mindfulness practices that meet individuals’ needs. It is suggested that brief mindfulness practice would likely be more feasible and time- and cost-effective for the general population than some longer mindfulness practices [23]. Norris et al. [23] showed that brief mindfulness practice improves executive attention even in naive practitioners, but only when controlling for neuroticism. Hence, it remains unclear whether brief mindfulness practice would be helpful to individuals with the neuroticism personality trait.

In studies on neuroticism, psychometric analyses based on self-report questionnaires and subjective assessments are mostly used as the assessment tools. In recent years, more and more studies have used objective measurement tools to complement psychometric analysis in order to better evaluate the effect of intervention training in neurotics [25,26,27]. Many studies were carried out to explore the unique psychological and neural processes involved in mindfulness practice based on other tools such as electroencephalogram (EEG) [28,29,30], functional magnetic resonance imaging (fMRI) [28], and electrodermograph [29], to name a few. It is also implicit that students can use technology to enhance their life quality through evaluating the impact of mindfulness practice. New research encourages the use of brain-sensing devices to improve cognitive functioning through mindfulness, e.g., [31] (for a review, see [32]). The attempt to identify an EEG channel to evaluate the effect of mindfulness is an ever-growing interesting research area. There are EEG devices that have been used to improve education through mindfulness with one [33] or more channels [34]. Using simple, relatively low-cost equipment may help neurotic students to evaluate the effect of mindfulness and encourage them to keep consistent practice, and therefore facilitate healthy life-style monitoring.

In this study, we investigated the effect of breathing-based mindfulness intervention (BMI) on neurotic students’ emotional self-regulation through psychological changes, physiological changes, and brain activities. However, in this paper, we will only present our psychological and EEG data analyses. EEG was used to record the students’ brain activities while psychometric tests were administered for psychological/behavioral analysis. EEG is arguably more feasible and cost-effective than MRI, which has the potential to induce claustrophobic anxiety amongst neurotic subjects. The EEG data acquired in our study were correlated with BMI using frequency analysis to investigate significant EEG changes potentially induced by improved ER, mediated through BMI in order to characterize the effects of BMI on ER. We were particularly interested to discover whether 5 min of brief daily mindfulness practice could improve the emotion self-regulation of neurotic students when experiencing an emotional situation induced by stimuli, and whether a single EEG channel was sufficient to identify this effect of BMI. We hypothesized that the effects of BMI, especially on the aspect of emotion self-regulation for neurotic students, could be observed and measured through EEG rhythms.

The paper outline is as follows. In Section 2, we provide the review of related works to further understand the relationship between neuroticism, emotion regulation, and mindfulness, and a review of EEG-based neural correlates of mindfulness. Next, Section 3 presents the materials and methodology of our work, and Section 4 discusses the results and presents the analysis of the results. Lastly, Section 5 concludes the paper with the research limitations and suggests further work in this area.

## 2. Related Works

### 2.1. Relationship between Neuroticism, Cognitive Bias, and Emotion Regulation

Neuroticism is also known as emotional stability, which refers to the capability of a person to remain stable and balanced in relation to their surroundings [35,36,37]. Individuals with a high-level of the neuroticism personality trait have a greater tendency to be negatively affected by emotions related to the limbic system [38] and more dispositionally inclined to mental conditions such as anxiety and depression than others [1,2,3,4], due to increased sensitivity to fear, anxiety, and distress [27]. They also tend to have difficulties in interacting with others due to their low emotional stability, which causes them to feel stress easily, have low self-esteem, and tend to have negative emotions. As they have a low threshold for negative emotions, they are more likely to respond to emotional stimuli [39]. Adolescents in the highest quartile of neuroticism (at age 14) were found to have two to three times higher rates of psychotic symptoms [40].

Low emotional stability in individuals with a high neuroticism level may be due to their difficulties in using emotion regulation (ER) strategies early in the process of emotion generation [41]. ER is a process whereby humans regulate their feelings and control them to avoid being guided or influenced by emotions, especially the negative ones. The vital part of emotion regulation is balancing positive and negative emotions, so that action taken later will have a valuable impact on people and surroundings [10]. There are two major strategies in ER [42,43]: cognitive reappraisal (CR) (adaptive) and expressive suppression (ES) (maladaptive). The adaptive ER strategies can help to change a person’s emotion towards taking the right action and staying focused on the present moment. Reappraisal is reframing the emotions of an experience in order to counter a problem in a positive manner rather than result in a negative reaction [16]. CR is reported to reduce anxiety by distinct cognitive control mechanisms [44]. On the other hand, ES represents a response-focused strategy that involves maintaining a neutral facial expression to hide one’s current emotional state [43]. ER strategies have been proven to have correlation with the neuroticism trait [43], particularly ES.

Cognitive bias/failure can be one of the factors that causes low emotion regulation. Cognitive biases (CBs) are subconscious errors in thinking that stem from problems related to memory, attention, and other mental mistakes that lead to the misinterpretation of information from the surroundings and affect the rationality and accuracy of decisions and judgments (due to faulty intuitions or improper analytical reasoning) [45]. CBs can occur at the level of attention, interpretation, or memory [46]. In the Big Five personality traits, neuroticism appears to be the only trait to be correlated to cognitive biases [47].

### 2.2. Mindfulness and Its Role as Mediator between Neuroticism, Cognitive Biases, and Emotion Regulation

Mindfulness has been found to significantly mediate the relationship between neuroticism and cognitive biases, while ER has been shown to mediate the relationship between cognitive biases and depressive symptoms (for a review, see [48]). It has been discovered that neuroticism has a negative correlation with mindfulness, meaning a person with a high neuroticism level is low in mindfulness, and vice versa. Mindfulness, which is known as an effective strategy for ER, can be defined as a quality of present experience/awareness; it is often practiced through meditation that involves an increased awareness of the surroundings, including emotions, thoughts, and feelings, accompanied by a sense of acceptance but without responding reactively, whether that be through physical symptoms, thoughts, or feelings [20,42]. Mindfulness has been used to keep a person’s mind in the present and fully engaged in/aware of the activities of the moment, which usually occurs through the reappraisal process. Mindfulness helps to improve adaptive actions when facing unexpecting obstacles by reducing the focus on worrying and being anxious and by increasing a sense of calm/equanimity [49] in order to find the proper solutions. The vital aspect of mindfulness is to help one shift from unguided attention (mind wandering) to becoming more aware and focused on the present, decreasing mental judgements and increasing the acceptance of negative emotions [50]. Nurturing the ability to focus on the present moment and dissociate from inner cognition or emotions that is embraced during mindfulness training can improve cognitive function and enhance ER strategies [51]. Zhang et al. (2019) stated that many studies have shown factual evidence for the effectiveness of mindfulness meditation in regard to ER [50].

There are many types of elements of focus used in mindfulness meditation practice that can suit various mental problems, with different minimum lengths of time required for each (or for multiple in combination), such as breathing (5 min), body scan (3 min), sound (3 min), self-compassion (9 min), and working with difficulties (7 min), to name a few [52]. Intermittent mindfulness practice was found to be associated with significant gains in wellbeing, trait mindfulness, and self-efficacy [53].

One of the elements of mindfulness, that is, paying attention to the present moment, is also one of the ER strategies used to balance emotional states. To reappraise negative emotions, attention must be withdrawn from the negative interpretation and redirected towards finding alternatives to handle the situation in the best way. Reappraisal is found to mediate the link between mindfulness and positive emotions, while suppression mediates the link between mindfulness and negative emotions [54]. Being attentive helps people to filter out unnecessary information, sensations, and perceptions and put all their energy into focusing on the controllable thing. This shows that being mindful without acknowledging irrelevant judgements can be very useful in reducing cognitive biases [45,46,47] and improving emotion regulation [42,49,50,51] to treat anxiety and depression [44,48].

Training in mindfulness meditation has been proven to reduce anxiety significantly in clinical [55,56] and experimental settings [44] (see the review in [57]). It is also claimed to be effective for a variety of psychological problems, not only in reducing/regulating symptoms of anxiety [10,44,58], but also those of depression and stress through the improvements achieved in attentional control that led to improved emotion regulation processes [10,58]. Several research studies provide evidence that the prefrontal cortex (PFC) and functionally-related structures mediate ER processes (for a review, see [59]). The role of emotion regulation in cognitive functions which are sensitive to both mindfulness and anxiety, i.e., cognitive conflict control and working memory capacity, was not taken into account in several related studies [57].

### 2.3. Neural Correlates of Mindfulness Using Encephalography

The evaluation of brain signals using EEG has been widely used to understand the mechanisms of human behavior, cognition, and emotion by measuring physiological changes in the brain in an objective, noninvasive, and continuous manner [30,31,32,33,39,60,61,62]. In contrast to neuroimaging tools such as the MRI scanner that could have the potential to induce claustrophobic anxiety amongst patients, EEG is arguably more feasible and cost-effective. It is a noninvasive, neurophysiological method of passively monitoring and recording electrical activity in the brain. Most of the activities in several parts and regions of the brain can be assessed and examined using EEG. The EEG spectrum can be decomposed into five specific wave bands/sub-bands following oscillation frequencies: delta (<4 Hz)—dreamless sleep state; theta (4–8 Hz)—meditation state; alpha (8–12 Hz)—relaxation state; beta (12–38 Hz)—active state; and gamma (38–42 Hz)—highly active state. The spectral analysis of EEG signals, which corresponds to the signal characterization in the frequency domain, was mostly used in the early studies on meditation. Among the brain waves, the alpha, theta, and beta waves have been recognized as important brain activity correlates of mindful awareness or, in short, neural correlates of mindfulness [29,30,61]. A substantive line of research has detected increased activity in the alpha and theta frequency ranges during mindfulness meditation and in the resting state following meditation (for a review, see [30,61]). On the other hand, a decrease in beta activity during mindfulness meditation has been extensively reported [61,62,63], which is potentially due to the reduced anxiety and stress achieved through mindfulness. The beta wave has been reported to emerge notably in people experiencing anxiety and stress due to external stimuli [57,61].

The neuropsychological mechanisms of mindfulness-induced changes in ER have also been studied using EEG by investigating neural oscillations/activities [62,63]. All these findings have been collectively thought to indicate internally directed focused attention [30]. It has been reported that alpha oscillations play a role in attention regulation, inhibition, information processing, memory consolidation, and the filtering of incoming sensory input from the environment. On the other hand, apart from memory consolidation, theta oscillations have also been correlated with inhibitory processes [31]. They also indicate a deep internally focused state connected to self-reflection. Findings in the alpha and theta wavebands from many comparisons made between the spectral signatures of meditation and those of other cognitive activities remain to be noticeable.

Among the most important aspects of anxiety disorders are deficits in the regulation and inhibition of negative emotions (for a review, see [59]). Increased delta and theta; decreased alpha, beta, and gamma bands; and more frequent transitions between these frequency bands are shown in spontaneous EEG activity in some anxiety disorders (for a review, see [61]). However, there is lack of studies exploring the improvement in ER achieved through brief breathing-based mindfulness to reduce anxiety among individuals with high neuroticism.

In neuroimaging studies, emotion regulation abilities that are associated with a set of prefrontal brain regions involved in cognitive control and executive functioning are shown to mature late in development. Hence, children and adolescents may have a harder time regulating their emotions [63]. However, since emotion regulation capacities can develop significantly across adolescence, treatment strategies need to be in place to help students with high neuroticism to improve their quality of life. Mindfulness meditation, which is a very flexible and simple (in regard to time, place, and mechanisms) yet effective for of meditation, can be one of the best options.

## 3. Materials and Methods

### 3.1. Participants

A total of 20 healthy female student volunteers were recruited from Universiti Teknologi PETRONAS (UTP). All-female participants were recruited as women are reported [62] to be more susceptible to mood and anxiety disorders [64,65], exhibit greater effects of ER [66,67], and have higher potential to adopt a meditation practice [68]. Furthermore, the implementation of a single gender in this study was also to reduce variances between participants and, therefore, identify accurately (properly) the effects of BMI, as this could eliminate the possible effect of gender difference on emotion elicitation and regulation. The individuals were identified as neurotics or belonging to the neuroticism group based on the scores of both the Big Five Inventory (BFI) [8] and the Eysenck Personality Inventory (EPI) [69] test. The individuals were aged between 18 and 25 years old, right-handed, and had normal or corrected-to-normal vision.

We excluded individuals who had hearing impairment (e.g., using hearing aids), were experiencing chronic mental stress or adverse psychological states (e.g., depression), or had a family history related to psychiatric or cognitive disorders. Individuals who were experiencing drug abuse or under medication during or within seven days before the study took place were also excluded from the study.

All recruited student volunteers provided written informed consent and were compensated for their time. This study was approved by the Medical Research Ethics Committee of UniKL Royal College of Medicine Perak, Malaysia (UniKLRCMP/MREC/2018/011).

### 3.2. Experimental Materials

#### 3.2.1. Personality Assessment

For the purpose of screening the participants, the BFI and EPI were used. The BFI test is a self-report inventory designed to measure the Big Five dimensions, which are openness to experience, conscientiousness, extraversion, agreeableness, and neuroticism, based on a 44-item multidimensional personality test that has proven to be a valid measuring tool of personality. On the other hand, the EPI questionnaire was developed to measure two biologically based independent dimensions of temperament: extraversion/introversion (E) and neuroticism/stability (N). The two questionnaires were used for screening participants’ neuroticism scale as both have a neuroticism measurement, which is the psychological trait this study is focusing on.

#### 3.2.2. Psychometric Tests and Measures

This section explains the psychometric tests administered in this study, which were based on self-report questionnaires/surveys. Self-report surveys were employed in this study because they are commonly used in psychological studies as they can yield much valuable and diagnostic information to a researcher or a clinician.

To assess the participants’ emotional condition and difficulties in emotion regulation, the Depression Anxiety Stress Scale (DASS) [70], Emotion Assessment Scale (EAS) [71], and Difficulties in Emotion Regulation Scale (DERS) [72] were used, respectively. DASS is a 42-item self-report instrument designed to measure three related negative emotional states of depression, anxiety, and tension/stress. EAS is a self-report test that divides emotional intelligence into the appraisal of emotion in the self and others, the expression of emotion, the regulation of emotion in the self and others, and the utilization of emotion in solving problems. DERS is a 36-item measure instrument to assess difficulties in emotional regulation. It can be used before and after a treatment to show progress in emotional regulation.

To quantify mindfulness, a commonly used 39-item five-facet mindfulness questionnaire (FFMQ) [73] that distinguishes five factors of mindfulness (observing, describing, acting with awareness, non-judging, and non-reactivity) was used. It was one of the first measures to evaluate the impact of mindfulness. These 4 questionnaires (EAS, DERS, DASS, and FFMQ) were administered prior to the experiment.

To assess participants’ emotional experience during tasks with stimuli based on video clips and Stroop task (refer to Section 3.2.3), Self-Assessment Manikin (SAM) [74] and NASA task load (NASA-TLX) [75] were used, respectively. SAM is a nonverbal pictorial assessment technique that measures the pleasure, arousal, and dominance associated with a person’s affective reaction to various stimuli. NASA-TLX, on the other hand, is a subjective multidimensional assessment tool that rates perceived workload in order to assess performance. It consists of six subjective subscales: mental demand, physical demand, temporal demand, performance, effort, and frustration. The NASA-TLX was used to assess whether or not the participants felt overwhelmed by a challenging task through the rating they gave.

Feedback from the participants upon completion of experiment/data recording was obtained through the Emotion Regulation Questionnaire (ERQ) [43]. ERQ is extensively used to evaluate two ER strategies (i.e., CR and ES) and the constant tendency to regulate emotions by CR or ES.

#### 3.2.3. Stimuli

##### Emotion Elicitation Video Clips

In this study, we selected emotional video clips as the stimuli to elicit/trigger upsetting emotions for the subjects. The descriptions of the video clips are given in Table 1. The video clips were selected from a well-established and validated study in emotion research [76]. In total, ten emotional video clips were used for the experiments to stimulate participants’ emotions: five in pre-intervention and another five in post-intervention. The condition of the participants when viewing these videos was thought to resemble their condition when encountering stressful life situations. The arousal value ranged from excited (9) to calm (1), while the valence value ranged from pleasant (9) to unpleasant (1). According to the Circumplex Model of Affect [77], the video stimulus is defined as ‘happy’ when the levels of valence and arousal are above 5. ‘Calm’ is when the level of valence is above 5, while the level of arousal is below 5. ‘Sad’ is when the levels of valence and arousal are both below 5. ‘Fear’ is when the level of valence is below 5, while the level of arousal is above 5. The participants were then asked to rate the video stimuli using the SAM scale with respect to affective valence (how pleasant or unpleasant) and arousal (the intensity of this pleasant or unpleasant experience) judgment. Even though different video clips were used before and after intervention phases, largely similar mean values of valence, arousal, and length were maintained, as can be seen in Table 1. The differences between the video clips in the two phases was insignificant, with *p* = 0.913, indicating high similarity between the videos. This was necessary to ensure the hypothesized change in participants was caused by BMI alone.

##### Color–Word Stroop Task

The participants were also asked to perform a 40-color-word Stroop task for 2 min. Visual stimuli based on the Stroop task were chosen as cognitive stimulation to put the participants in the condition of receiving day-to-day demanding tasks and to evaluate the effect of the induced negative emotions on participants’ cognition and attention span by examining a person’s ability to separate word and color-naming stimuli [78]. The Stroop task is one of the most well-known psychological experiments, named after John Ridley Stroop [79]. The Stroop effect is the delay in reaction time between responding to congruent and incongruent stimuli. The Stroop effect demonstrates that it is difficult to name the font color when there is a mismatch between the word meaning and the font color, which is termed incongruent. The test consists of 4 words, i.e., red, blue, green, and yellow, written with ink in the same color (congruent) or a different color (incongruent), and participants are required to provide the correct color of the ink in the specified time (2000 ms in this experiment). After performing the Stroop task, the participants were asked to rate their performance using NASA-TLX for 6 s.

#### 3.2.4. Breathing-Based Mindfulness Intervention (BMI)

A 6-week breathing-based mindfulness intervention (BMI) with a 1 h introduction to and training in mindful breathing (MB) was the intervention employed in this study. The MB technique, namely the 4-7-8 mindful/conscious breathing technique [80], can be described as follows: participants inhale mindfully from their nose for 4 s using counting to disturb ideas. Then, they let their thoughts raise and fall on their own accord and be at one with the participants’ breath. The participants hold their breath for about 7 s. Finally, the participants exhale slowly from their mouths, making a whoosh sound for 8 s. The 4-7-8 technique forces the mind and body to focus on regulating the breath to gain control over the breathing, while guiding one’s attention to the present moment rather than replaying worries.

Additionally, the subjects were also instructed to let their thoughts, emotions, or feelings come and go without making any judgement or reacting to them. The focus on breathing, which improved their recognition of awareness, helped them be nonjudgmental without much force. The participants were instructed to perform at least 5 min of MB every day but were encouraged to increase the time and practice whenever and wherever possible. Fourteen out of twenty students reported practicing MB for more than 5 min at different times of day, such as before sleeping, before starting classes, during stressful moments, and while walking back to their rooms after classes.

### 3.3. Study Procedure

**Figure 1:**
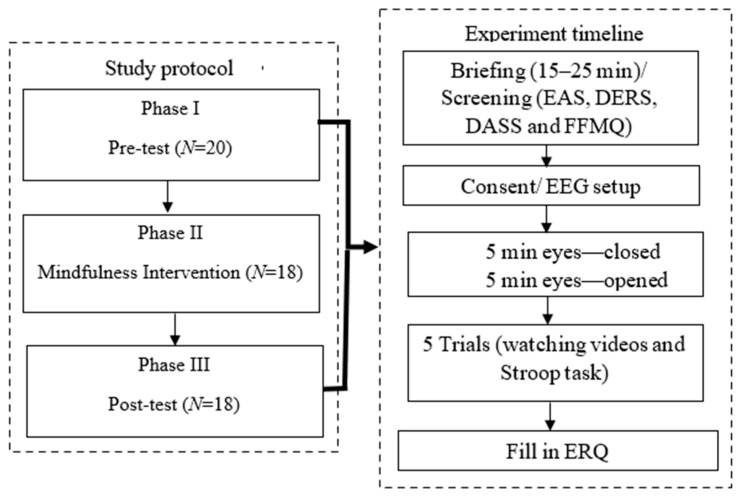
Flowchart of the study protocol and the experiment timeline.

Figure 1 depicts the flow of the entire study procedure. Student volunteers who met our recruitment criteria were briefed on the purpose of the study and their involvement during the experiment, including the risks and affects that might occur. The briefing was carried out prior to the start of data collection and after consent was obtained. The participants also needed to complete some screening tests, as described in the previous section, namely the EAS, DERS, DASS, and FFMQ, at least a few days before the experiment (in both phases I and II). These tests were given to provide us with ideas about participants’ emotional condition status.

The rest of the psychometric tests were administered as described in Section 3.2.2. Data acquisition was conducted twice: (1) pre-intervention (i.e., phase I and II) and (2) post-intervention (i.e., phase III). Phase II consisted of the breathing-based mindfulness intervention (BMI) training and practice. These phases are described further in the following sections.

#### 3.3.1. Experiment—Phase I (60–90 min)

**Figure 2:**
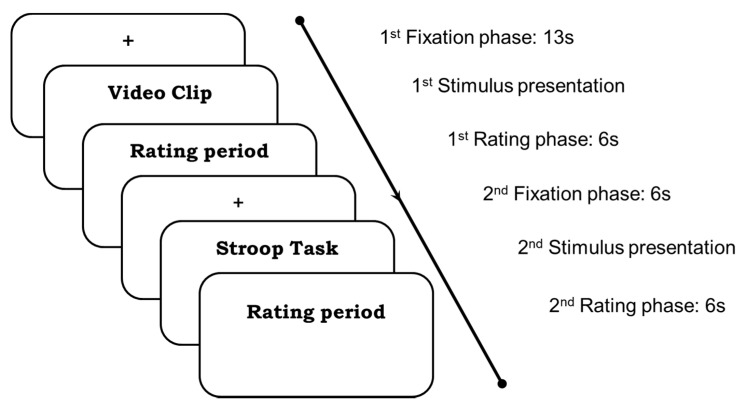
Schematic illustration of an experimental trial.

In phase I, we acquired pre-intervention data, including the recording of the EEG and physiological responses of the participants. Figure 1 depicts the experiment block showing the timeline of the experiment. The experiment began with 5 min eyes-opened (EO) followed by 5 min eyes-closed (EC) sessions, which served as baseline/resting condition, i.e., data of participants in neutral condition without task-demand brain activities. This was a necessary step to account for subjective variability. For EC, subjects were instructed to close their eyes while staying awake, whereas for EO, subjects were instructed to gaze at a fixation point that appeared in the middle of the screen. The baseline condition was followed by 5 trials, and each trial had two consecutive stimuli: a video clip and a Stroop task. Figure 2 illustrates the procedure involved in each trial.

As can be seen in Figure 2, each trial began with fixation for 13 s to enable synchronization with physiological sensors and at the same time to get rid of the first 10 s that might contain noisy data of participants getting ready (or performing mindful breathing in Phase III). This was followed by the viewing of a video clip with a mean duration of 2.35 min (refer to Table 1). Then, participants performed the SAM rating for 6 s to evaluate their affective valence and arousal levels experienced during the watching of the video clips. Next was another 6 s of fixation, followed by a Stroop task to evaluate the effect of the induced negative emotions on participants’ cognition for a duration of 2 min. Finally, the participants were instructed to carry out another 6 s rating using the NASA-TLX to rate their experience attempting the Stroop task. Both the SAM and the NASA-TLX ratings were used to assess participants’ emotional experiences. Hence, with one video and a Stroop task in each trial, we had a total of 5 trials for the experiment in phases I and III.

In summary, each experiment had a baseline followed by 5 trials, where each trial consisted in fixation for 13 s followed by a single video clip viewing followed by a 6 s SAM rating and 6 s fixation followed by 2 min of Stroop task followed by 6 s task rating. To avoid participants experiencing fatigue, they were given a few minutes as a break between each trial. Participants’ feedback on their abilities to regulate their emotion during video clip viewings was also obtained upon completion of the experiment through ERQ. This experimental procedure was performed in the pre-intervention (prior to BMI) and post-intervention (after BMI) phases. At the end of the pre-intervention data recording, the instructor explained and distributed information and instructions related to the mindfulness intervention that would be carried out in phase II.

#### 3.3.2. Experiment—Phase II (6 Weeks)

In phase II of the study, all participants underwent mindful breathing (MB) training that lasted for 6 weeks. The study took place during the study semester. All the sessions were run during the study semester and participants had a full study load. In the MB training, the participants had to attend an introduction session in group format as the kick-off of the intervention phase, which was conducted by a medical psychiatrist from the RCMP, UniKL, for a duration of 1 h.

Upon completion of the introductory training session, the participants were given instructions on the 5 min daily practice of an MB exercise that they had to adhere to. More details on the MB exercise can be found in Section 3.2.4. During the study, all participants were added to a WhatsApp group created to send them daily reminders for the individual MB exercise. This was performed once a day to ensure participants’ adherence to the MB exercise and with minimum possible distraction. Furthermore, participants did not report any external activities that may have interfered with their MB exercise. Videos of the correct practice of MB, including the recorded inhouse introduction session, were provided to the participants. The subjects kept on being reminded to watch the recorded training session from time to time, especially in the first 2 weeks of their intervention period, so that their understanding and MB practice and outcomes could improve.

Every two weeks, they were required to attempt the Mindfulness Process Questionnaire (MPQ) to assess their practice of mindfulness and their experience throughout the 6 weeks of BMI in phase II. The MPQ was developed in such a way as to allow participants to assess their attempt at being mindful or engaging in mindfulness (7 items rated on a four-point Likert-type scale) rather than how successful they were at being mindful.

#### 3.3.3. Experiment—Phase III (40–60 min)

Once the 6-week intervention (BMI) was over, participants were called to come back for data recording for phase III, with a similar procedure to that used in phase I. The video clips were changed in phase III, while a similar mean of valence, arousal, and length was maintained (refer to Table 1). This was necessary to ensure that the hypothesized improvement of ER ability was not affected by repeating the stimulation.

The difference between the video clips in the two phases was insignificant, with *p* = 0.913, indicating high similarity between the videos. This was necessary to ensure the hypothesized change between students before and after the intervention was caused by the BMI itself.

Before and during the experiment/data acquisition in phase III, participants were asked to perform MB as comfortably as possible. By phase III, they should have been very familiar and comfortable with performing mindful breathing at their own pace.

### 3.4. Methods

#### 3.4.1. EEG Data Acquisition

An Enobio^®^ 20-channel dry sensor EEG system with a wearable wave guard cap was used to measure and record the brain activity signal of the participants during the experiments. Dry electrodes were used to ensure comfortability of participants while maintaining good signal quality before proceeding to experiment. The EEG electrodes were set up based on the 10–20 international system. The reference electrodes of common-mode sense (CMS) and driven right leg (DRL) were placed on the right mastoid. In addition to EEG, the participants’ peripheral physiological responses, namely the skin conductance (SC), blood volume pressure (BVP), respiration rate/heart rate (HR), and temperature (T), were also observed and recorded using the FlexComp Infiniti encoder with Biograph Infiniti software by Thought Technology. The sensors for measuring SC, T, and BVP were placed on their left hand, and the HR belt was placed around their chest.

#### 3.4.2. EEG Data Analysis

EEG data helped identify the brain regions affected by mindfulness. The EEG recording, as described earlier, was performed two times, in phase I (before BMI) and in phase II (after BMI). Each student recorded a baseline and 5 trials: EC and EO, and five short videos to induce negative emotions followed by a Stroop task, respectively.

**Figure 3:**
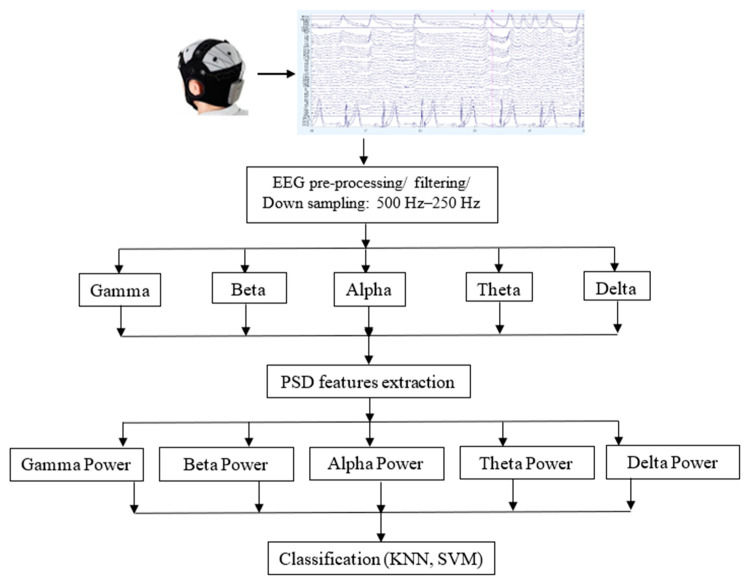
General framework.

To study the change of EEG data, the signal power was obtained using the flow described in Figure 3. The raw EEG data were filtered according to respective frequencies gamma, beta, alpha, theta, and delta. The raw EEG data were preprocessed using BESA Research 6.0 for artifact correction. The drifts in EEG obtained were corrected, and a notch filter of 50 Hz was applied to remove line noise. The data were then filtered using FIR filter with a low frequency of 0.5 Hz and a high frequency of 47 Hz. Then, the data were downsampled from 500 Hz to 250 Hz, because this provided an accurate analysis within half of the analysis time required for 500 Hz frequency. Then, the cleaned EEG data were exported to MATLAB and frequency analysis was performed.

The signal power for each wave band (gamma, beta, alpha, theta, and delta) was calculated using the periodogram method by Welch [81], through a Hanning window function [82]. First, the EEG data were segmented into eight segments with 50% overlap. The Hanning window was then used to compute the modified periodogram of each segment. Power Spectral Density (PSD) was then calculated for each of these segments. After that, the average PSD was calculated for all the segments to obtain the absolute power for each wave band by log transformation using the natural log and then multiplying the value by 10.

Equation (1) shows the average of the squared Fourier transform of the EEG signal. Denoting the *m*th windowed frame from the signal *x* by

*x_m_*(*n*) = *w*(*n*) *x*(*n* + *mR*), *n* = 0, 1, …, *M* − 1, *m* = 0, 1, …, *K* − 1,

where *R* is the window hop size; the periodogram, *P* of the *m*th block and *K* number of frames can be written as:

(1) $$P_{x_{m},M}\left(w_{k}\right)=\frac{1}{M}{\left|\sum_{n=0}^{N-1}x_{m}\left(n\right)e^{-j\frac{2\pi kn}{N}}\right|}^{2}$$

Then, the Welch estimate of the PSD can be obtained by averaging the periodogram of the successive blocks as the following:

(2) $${\hat{s}}_{x}^{w}\left(k\right)=\frac{1}{K}\overset{K-1}{\underset{m=0}{\sum }}P_{x_{m},M}\left(w_{k}\right)$$

The filtering process was performed to separate the EEG signal into the well-defined bands then the PSD was calculated for each band separately to show the strength of the variation of the signal (energy distribution) as a function of frequency. The calculated EEG power of each wave was then averaged across brain regions, i.e., parietal (P7, P4, Pz, P3, and P8); frontal (Fp1, Fp2, F3, F4, Fz, F7, and F8); temporal (T7 and T8); central (Cz, C3, and C4); and occipital (O1, O2, and Oz).

#### 3.4.3. Classification

To gain better insight into the impact of BMI on students’ brain activities, we used the KNN and SVM algorithms to evaluate the classification (pre- or post-BMI) performance of the channels with the most significant changes between pre- and post-BMI. KNN is one of the simplest classification algorithms that compares a data point to its neighbors. Then, it groups the data point to a class based on the shortest distance to its neighbors. For the KNN, we used a standardized model consisting of the Euclidean distance function with equal-distance weights. SVM works by creating a hyperplane to separate data points into different classes. It was used because it can work for linear as well as nonlinear problems. We employed a linear kernel due to its efficiency in similar studies [83].

The data of three subjects in the eyes-opened session and five subjects in the eyes-closed session were excluded from the analysis because of technical errors that occurred during EEG recording. These errors were not related to the subjects’ behavior, but were caused by either faulty EEG data embedded with DC components or a corrupted EEG file, and therefore were not appropriate for analysis. Hence, the PSD features of the pre-frontal Fp1 channel from 13 subjects during 5 min eyes-opened and the PSD features of the occipital lobe O2 channel from 15 subjects during 5 min eyes-closed sessions were fed into the classifiers. Ten-fold cross-validations using the KNN and SVM algorithms are compared and discussed in the following section.

## 4. Results and Findings

The results and findings of this study can be divided into two main parts of the data analysis. The first is based on the behavioral data, which refers to the results from the psychometric tests/questionnaires and the visual stimuli administered before and after the experiment in both phases I and III, namely the AES, DASS, FFMQ, and ERQ. The latter is based on the analysis of the brain activities acquired using EEG. The analysis of the peripheral physiological data is not presented in this paper.

It is to be noted that 18 out of the 20 subjects successfully completed all three phases, and therefore their questionnaire data were included in the following behavioral data analysis. However, for the EEG analysis, due to technical errors (as explained previously), some trials for some subjects were excluded because of corrupted files or incomplete records. Hence, for the sake of clarity, the number of data used for each analysis is mentioned in the respective section.

### 4.1. Behavioral Data Analysis—AES, DASS, FFMQ, and ERQ Questionnaires

The behavioral data analysis discussed in this section is based on the results of four questionnaires, namely the AES, DASS, FFMQ, and ERQ, administered in phase I (pre-intervention) and phase III (post-intervention). To evaluate the changes in the results of the questionnaires before and after BMI, repeated measures analysis of variance (ANOVA) was used. The sample size in this analysis was 18. As explained previously, 18 out of the 20 subjects successfully completed all three phases of this research, and therefore their questionnaire data were included in this behavioral analysis.

Table 2 shows the results from the AES, FFMQ, and DASS questionnaires, which were administered prior to the start of the experiment in phase I (before BMI) and phase III (after BMI). The first questionnaire was an assessment of emotion scale (AES), which gave us an idea of the emotional intelligence of the participants. The score of this questionnaire can range between 33 and 165, with higher scores indicating more emotional intelligence. Repeated measures ANOVA showed no significant difference in emotional intelligence before and after BMI: *F*(1,17) = 0.975, *p* = 0.337, as summarized in Table 2. On the other hand, it can be observed that the results of the five-facet mindfulness questionnaire (FFMQ) showed a significant change in the students before and after BMI in two facets of mindfulness, i.e., non-judging and non-reactivity to inner experience: *F*(1,17) *=* 4.95, *p =* 0.04 and *F*(1,17) *=* 7.79, *p* = 0.013, respectively. These significant results indicate that BMI helped students be less judgmental and non-reactive to inner experience (feelings, thoughts, situations, and emotions), which impacted their calmness and gave rise to better, clearer thinking. This could also indicate the improvement of mindful ER among the students [20,42].

The next questionnaire, the depression, anxiety, and stress scale, or DASS, showed a significant decrease in the mean scores (before and after BMI) for anxiety (*F*(1,17) = 7.61, *p* = 0.013) and for stress (*F*(1,17) = 5.89, *p* = 0.027). This could support the improvement in ER achieved by the students, as enhanced ER strategies have been proven effective in treating anxiety and depression [44,48]. No significant change in the depression scale was observed, with *F*(1,17) = 0.63, *p* = 0.438. We saw a large difference in the mean scores obtained for anxiety and stress levels but not for depression (difference of <2), which could be the reason why there was no significant reduction in depression level reported.

Finally, to assess students’ emotional regulation after completing the experiment in phase I and phase III, the emotion regulation questionnaire (ERQ) was administered. The results tabulated in Table 3 show the significant effect of BMI on the students’ emotion regulation strategy, that is, suppression (ES). After BMI, students showed a significant decrease in the suppression of emotions: *F*(1,17) = 4.620, *p* = 0.046. On the other hand, the mean score of appraisals as a way of regulating emotion increased (insignificant, *p* > 0.05) after BMI. Appraisals mediate between mindfulness and positive emotions; the increase in the mean score shows that the students improved their reappraisal strategy of ER, which is positively related to wellbeing, whereas using suppression is negatively related [43]. These results further convinced us of the improvement in mindful ER achieved by the students. Even though the video stimuli used in the experiment were intended to trigger upsetting emotions, the subjects were shown to be able to regulate their emotions without the extensive use of the ES strategy. This also suggests that students are more open and able to experience their emotions without suppressing them after BMI, which is one of the main benefits/effects of mindfulness meditation.

### 4.2. EEG Data Analysis

Among the EEG channels used in the experiment, some channels were more significant than others in detecting BMI’s effect. The significant channels were those that showed significant changes in the mean of the PSD (<0.05) of the brain activities between the two conditions (before and after BMI). This analysis was conducted by using the paired *t*-test. The selected significant channels were then used as features to evaluate their correlation to the effects of BMI using machine learning models. More details on this significant channel analysis and classification can be found in Section 4.2.1 and Section 4.2.2, respectively.

#### 4.2.1. Significant Channel Analysis Using PSD

In this section, we will present the significant channel analysis of the acquired EEG data. Among the EEG channels used in the EEG recordings, some channels were more significant than others in detecting the changes potentially induced by BMI. The spectral analysis showed significant changes in some channels between the two conditions (before and after BMI). Table 4 shows the significant channels that correlated with the effect of BMI during baseline and several tasks, e.g., EC and EO, for different wave bands. This result depended on the analysis provided by the paired t-test to compare the means of the PSD of the brain activities between students in the two conditions.

To address closely the changes between the students before and after BMI, as stated earlier, the PSD was calculated for each channel over the scalp and was categorized according to each hemisphere and lobe separately. The results were averaged across the brain regions, i.e., the parietal region was the average of P7, P4, Pz, P3, and P8; the frontal region was the average of Fp1, Fp2, F3, F4, Fz, F7, and F8; the temporal region was the average of T7 and T8; the central region was the average of Cz, C3, and C4; and the occipital region was the average of O1, O2, and Oz.

Overall, from Table 4, we can see that the Fp1, O2, F3, F4, and T8 channels were identified as significant channels. This is in line with the findings from [84], which concluded that electrodes F3, F4, and T8 had the best effect on emotion classification, while another study [85] showed that the prefrontal lobe and occipital lobe greatly contribute to emotion classification. Furthermore, Fp1 and F3 are both in the left hemisphere, which could indicate an increase in approach motivation [86], a condition for goal-directed behavior that could be achieved through mindfulness, as neurotic subjects tend to have low approach motivation.

It is also noticeable that when the eyes were closed, the occipital region (O1 and O2) had the most significant difference between the students before and after BMI, especially in the alpha and theta rhythms, indicating that the students were relaxed while their eyes were closed. On the other hand, the frontal channels were observed to be more active during the eyes-opened trials and during the stimuli viewing, especially for Fp1 in the delta and theta bands, indicating greater eye movement. The spontaneous eye-blink rate indicates the presence of the neurotransmitter dopamine in the striatum, which is associated with arousal, reward-driven learning, motivational signaling, and, most relevant to the present study, attention [87].

We further narrowed down the identified significant channels to the significant channels that were most-often identified across wave bands and across tasks, as presented in the last column and the last row, respectively, in Table 4. As can be observed, we found that Fp1 had significant differences in the delta rhythm during EO, Trial 3, Trial 4, and Trial 5, while the occipital region channel O2 had a significant difference in the theta, alpha, and beta rhythms during the EC session. These findings agree with the claims that alpha, theta, and beta have been recognized as important brain-activity correlates of mindful awareness (see Section 2.2). Hence, the Fp1 and O2 channels were selected as the most significant channels, which could potentially be used to identify the effect of mindfulness. We then evaluated the classification performance of the Fp1 and O2 channels as EEG features for identifying the effects of BMI.

#### 4.2.2. Classification

**Figure 4:**
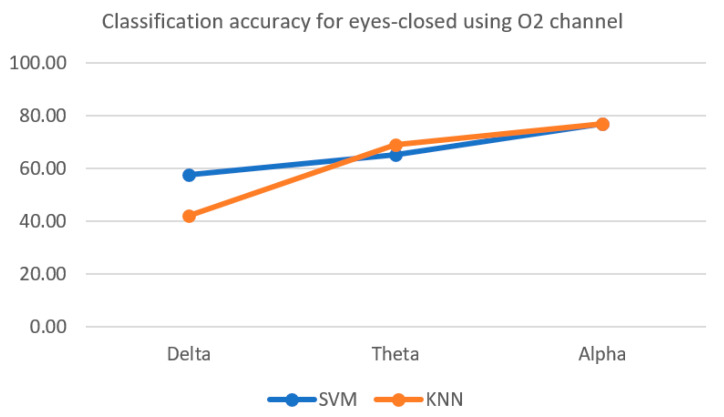
Classification accuracy using O2 channel during EC for delta, theta, and alpha wave bands.

As described in the methods section, the KNN and SVM classifiers were applied to the EEG features. The results were compared for different EEG rhythms. Figure 4 shows that the alpha band performed better in separating the two classes during the EC session compared to the other two brain waves.

This could be due to alpha waves being well-known for being a universal sign of relaxation. On the other hand, the delta rhythm achieved the lowest accuracy. This is because it is expected to be activated for the subject when the eyes are closed. Furthermore, in the delta frequency band, the classification accuracy using the O2 channel achieved by KNN was lower than that of the accuracy achieved by SVM. However, the KNN accuracy was slightly higher than that of SVM in the theta frequency band. In the alpha frequency band, both algorithms had nearly identical classification accuracy.

Table 5 summarizes the classification performance of the KNN and SVM algorithms in terms of accuracy, sensitivity, specificity, and area-under-the-curve (AUC) obtained for the O2 channel on the alpha band. Ten out of the thirteen subjects were correctly classified as either pre-BMI or post-BMI, and this explains the similarity of the scores of the sensitivities and specificities obtained. The AUC values indicated that the KNN classifier slightly outperformed SVM by about 0.05. However, both classifiers had the same good accuracy, indicating O2 as a potential channel for identifying the effect of mindfulness. The accuracy obtained in this research (76.9 ≈ 77%) using a single EEG channel was similar to the accuracy obtained (78%) in [88] using 32 EEG channels for differentiating between subjects under mindfulness meditation and a control group.

As explained previously in Table 4, the delta rhythm showed the potential of the Fp1 channel to differentiate between the two conditions when the eyes are opened or during trials. A similar performance was obtained with 15 subjects, as shown in Table 6. However, SVM slightly outperformed KNN, as the value of the AUC (as a cross-validation parameter) indicates. The difference in performance between the two classifiers in both channels, however, was insignificant, as shown in Table 7, suggesting that any of the classifiers could be used efficiently to classify BMI. However, SVM is the most-frequently used classifier in emotion recognition, according to the authors of [89]. Furthermore, SVM based on PSD as frequency domain features is reported to be the most-commonly used method in emotion classification [90].

The classification results obtained correlate well with the outcomes of the significant channel analysis presented in Table 4.

Although the classifier worked comparably to other studies, e.g., [88], further research is needed to validate the classifier with a larger sample size. In particular, it is necessary to demonstrate that the single channels Fp1 and/or O2 have the potential to successfully identify the effect of mindfulness.

## 5. Discussion and Conclusions

This research was conducted to evaluate the effect of brief BMI on improving emotion regulation among female students with neuroticism. Neuroticism is a personality trait that includes a lack of emotional stability, which negatively influences the lives of neurotic students. Therefore, this research represents a way to help the neurotic population improve their ability to regulate negative emotions by practicing brief mindful breathing. In this study, we observed through the changes in the EEG and behavioral data analysis that the negative emotions induced by watching short video clips (stimuli) were able to induce changes in EEG and psychological states. Through FFMQ, we could observe that the students’ levels of the non-judgement of inner experience and non-reactivity to inner experience decreased significantly after the intervention, with *p*-values of 0.04 and 0.13, respectively. This meant that through BMI, a significant improvement in students not being judgmental or non-reactive to their own inner experiences could be achieved. These two facets of mindfulness were considered important elements of mindfulness-based stress reduction (MBSR) regarding cognitive flexibility [10]. This is also an indicator that the improvement of ER is mediated by mindfulness, since non-judging and non-reactivity are reported to be achievable by improving attentional control or increasing the quality of present experience/the awareness of surroundings, accompanied by a sense of acceptance but without judgment or responding reactively [20,42].

Through the behavioral and EEG data analyses, we could also observe that the participants regulated their emotions better after 6 weeks of brief BMI, as the DASS questionnaire showed the significant effects achieved on reducing the students’ anxiety and stress levels, with *p*-values of 0.013 and 0.027, respectively, suggesting an improvement in the quality of life. A decrease in depression could also be observed, even if this decrease was not significant. This further supported the improvement in emotion regulation (ER) among the students, as ER strategies have been extensively reported to be able to balance emotional states. Furthermore, mindfulness, which is known as an effective strategy for ER, has also been proven to reduce anxiety significantly in clinical and experimental settings (refer to Section 2.2).

Moreover, the EEG analysis showed that there were significant differences between the students before and after BMI (*p*-values < 0.05) in resting states (e.g., eyes-closed and eyes-opened) and tasks (e.g., video stimulation and the Stroop task). These results support the EEG studies that reported significant differences between university students before and after mindfulness, represented by an increase in the alpha and theta and a decrease in the beta rhythms [34]. However, our study accounted for neuroticism, which has been suggested as a significant factor for studying the impact of mindfulness [23]. The present research proposed the use of single channels, i.e., O2 and Fp1, to classify pre- and post-BMI conditions during eyes-opened and eyes-closed baseline trials. The potential of these channels, especially with respect to ER, was displayed by a good performance in terms of accuracy (~77%), sensitivity (76–80%), specificity (73–77%), and area-under-the-curve (AUC) (0.66–0.8) obtained by the k-nearest neighbor (KNN) and support vector machine (SVM) algorithms. Dennis and Solomon [91] suggested that frontal EEG activity reflects the emotional context and emotional regulation capability, which supports further the potential of Fp1 to be used to identify the impact of BMI, especially for neurotic individuals. Further studies can be built upon the results from this study to evaluate other mindfulness-based intervention approaches using psychological and electrophysiological measurements to improve the quality of life of neurotic students.

Furthermore, through the ERQ, conducted after the experiment in phase I and phase III, BMI was also shown to be able to produce a significant effect (i.e., decrease) on the students’ ER strategy, that is, expressive suppression (ES), with a *p*-value of 0.046. This indicates that mindful breathing exercises helped the students release and express their emotions rather than suppressing them, even though the ES strategy has been proven to have a correlation with the neuroticism trait [43]. Students who performed mindful breathing had less suppression of their emotions, which was shown to have a positive impact on their health and inner peace, as can be seen in the outcome of their interventional studies. Furthermore, John and Gross (2004) provided new evidence toward a healthy emotion regulation profile, i.e., increases in the use of reappraisal and decreases in the use of suppression, which were observed in this study [41]. This is also in line with previous research showing that short mindfulness meditation improved emotion regulation [21,24,51,92]. It is thus feasible that such mindfulness be practiced by students led/reminded by their lecturers at beginning of class in either physical or virtual classroom settings, since this promotes a reduction in anxiety and cognitive biases that could positively affect students’ attention and focus on their learning and participation in class.

Although this study used a single-group pre–post design and employed multiple measures (self-report, EEG, and physiological responses) to account—to some extent—for possible biases in the self-reported data, it is recommended to include a control group to confidently attribute the changes before and after BMI to mindfulness alone. Furthermore, the relatively small sample size (18 subjects) and the recruitment of female participants might be another limitation in this study. Female participants were recruited to reduce the possible variation in the results. This was because several studies suggested possible differences between males and females in terms of emotional regulation [66,67,68] and mindfulness practice and effectiveness [93,94,95]. For example, the study of Rojiani et al. [95] showed greater decreases in negative affect and greater increases in scales measuring mindfulness and self-compassion for females compared to males in response to school-based mindfulness. Similar results were obtained by Kang et al. [93] from 100 participants, of which 46 were females. The study also suggested that gender-specific modifications to mindfulness-based intervention might maximize its benefits and effectiveness. Future research is encouraged to include both male and female participants with a larger sample size to replicate the results of our study, improve our perception, and provide a better understanding of this effect. Another limitation to this study is the lack of a follow-up with participants after the study. Knowing that several studies have found a positive correlation between the daily practice of mindfulness and self-esteem and self-acceptance, and a negative correlation between mindfulness and neuroticism, e.g., [96], it is encouraged to assess long-term mindfulness intervention and whether it can decrease the neuroticism trait.

In conclusion, this study is one of the few to investigate the effect of mindfulness on neurotic students by employing emotion regulation and EEG measures. The promising results of this research suggest the potential of single channels, i.e., O2 and Fp1, for eyes-closed and normal activities, respectively, to identify the effect of brief BMI on neurotic students’ emotion regulation ability, which can be observed in the outcomes of behavioral data analysis based on self-report data and EEG data analysis. However, future studies are strongly recommended to replicate these results and determine their robustness.

## Figures and Tables

**Table 1:** Mean values of emotional video clips used during experiment in the pre- and post-intervention phases.

Phase	Mean Length (min)	Mean Arousal	Mean Valence
Pre-intervention	2.35	5.26	2.73
Post-intervention	2.42	5.35	2.77

**Table 2:** Statistical comparison of results of pre- (T1) and post-intervention (T2) for AES, FFMQ, and DASS pre-experimental questionnaires.

Questionnaire	M(SD)		*F*-Value	*p*-Value	η^2^
	T1	T2			
AES	123.22 (11.53)	119.89 (9.65)	0.975	0.337	0.054
FFMQ-observe	28.72 (3.97)	27.787 (4.86)	0.702	0.414	0.40
FFMQ-describe	22.83 (6.4)	23.78 (4.15)	0.545	0.470	0.031
FFMQ-act-with-awareness	22.56 (4.95)	22.39 (4.62)	0.023	0.882	0.001
FFMQ-non-judgmental	17.17 (5.06)	19.33 (4.54)	4.95	**0.040**	0.225
FFMQ-non-reactive-to-inner-experience	19.94 (3.35)	22.33 (2.33)	7.79	**0.013**	0.314
DASS-anxiety	21.78 (6.36)	15.78 (8.51)	7.61	**0.013**	0.309
DASS-stress	21.33 (8.62)	16.11(6.42)	5.89	**0.027**	0.257
DASS-depression	13.33 (7.7)	11.56(7.66)	0.63	0.438	0.036

**Table 3:** Statistical comparison results of pre- (T1) and post-intervention (T2) for post-experiment emotion regulation questionnaire (ERQ).

Q	M(SD)		*F*-Value	*p*-Value	η^2^
	T1	T2			
ERQ-appraisal	29.56 (4.31)	30.89 (3.72)	1.766	0.201	0.094
ERQ-suppression	18.17 (3.67)	15.89 (3.67)	4.620	**0.046**	0.214

**Table 4:** The significant channels obtained from paired *t*-test analysis of brain activities between students in the two conditions (pre- and post-BMI) that could serve as potential EEG channels to identify/indicate BMI effect.

Brain Wave	Eyes-Closed (EC)	Eyes-Opened (EO)	Trial 1	Trial 2	Trial 3	Trial 4	Trial 5	Most-Selected Channel
Delta	-	Fp1, F3	P4	-	P4, O1, Fp2, Fp1	P3, Fp1, T7	Fp1, Fp2, P4	Fp1 ***
Theta	P8, O1, O2	F4	-	-	-	-	FP1, F4	F4 **
Alpha	O1, O2, T7	F3	Pz	-	-	-	C4, F4, C3, F3	F3 **
Beta	O2	-	-	-	T8	-	-	NA
Gamma	-	-	-	-	T8	-	-	NA
Most-selected channel	O2 ***	F3 **	NA	NA	T8 **	NA	Fp1 ** F4 **	O2 ***, Fp1 ***

** Indicates two times appeared as significant channel; *** indicates three times appeared as significant channel; NA—appeared as significant channel less than twice.

**Table 5:** Evaluation of classification performance for O2 channel.

Classification Method	Accuracy	Sensitivity	Specificity	AUC
KNN	76.9%	76.9%%	76.9%%	0.8
SVM	76.9%	76.9%%	76.9%%	0.75

**Table 6:** Evaluation of classification performance for Fp1 channel.

	Accuracy	Sensitivity	Specificity	AUC
KNN	76.7%	80%	73.3%	0.66
SVM	76.7%	80%	73.3%	0.75

**Table 7:** Classification performance for O2 and Fp1 channels achieved by both the KNN and SVM classifiers.

Channel	Accuracy	Sensitivity	Specificity
O2	76.9%	76.9%	76.9%
Fp1	76.7%	80%	73.3%

## Data Availability

Data available on request due to privacy/ethical restrictions.
